# Seroconversion of hepatitis B surface antigen among those with previously successful immune response in Southern China

**DOI:** 10.1080/21645515.2020.1801076

**Published:** 2020-09-22

**Authors:** Zhigang Zheng, Guojian Li, Fuhui Liao, Lujuan Zhang, Xueyan Wang, Zhongliao Fang, Qinyan Chen, Huabin Liu, Liping Hu

**Affiliations:** a Department of Epidemiology and Statistic, School of Public Health, Guangxi Medical University, Nanning, China; b Guangxi Key Laboratory for the Prevention and Control of Viral Hepatitis, Guangxi Zhuang Autonomous Region Center for Disease Prevention and Control, Nanning, China

**Keywords:** Hepatitis B vaccine, seroconversion, HBsAg, immunization cohort, Long An County

## Abstract

Recommendations promoted worldwide have suggested a period of protection lasting more than 20 years against hepatitis B (HB) following primary immunization. Starting in 1987, universal HB vaccinations were carried out in Long An County, Guangxi Province, one of the earliest counties in which plasma-derived HB vaccine was delivered to newborns across China. Data collection targeted toward understanding the long term (26–33 years since primary immunization) immune effects of the plasma-derived HB vaccine was conducted in 2015; a second data collection was carried out in 2019 to assess seroconversion in the same cohort. This study qualitatively compared positive vs negative results and quantitatively assessed HB biomarkers – HB surface antigen (HBsAg), antibody to HBsAg (anti-HBs), HB e-antigen (HBeAg), antibody to HBeAg (anti-HBe), and antibody to HB core antigen (anti-HBc) – in serum 26–33 years after the full initial course of HB vaccination, then analyzed anti-HBs seroconversion using the two-phase sampling method in the same cohort and calculated the anti-HBs seroconversion rate from 2015 to 2019. The protective sero-conversion rate (anti-HBs ≥10mIU/mL) was 37.6% (192/511); the HBsAg-positive rate was 5.3% (27/511); the anti-HBs mean geometric titer (GMT) was 11.1 mIU/mL. Among the 143 participants involved in both 2015 and 2019 data collections, the seroconversion rate was 3.5% (5/143); two individuals had protective anti-HBs levels in 2015. These findings indicate that anti-HBs status can be seroconverted to a protective concentration level 4 years earlier in a high HBV epidemic region. The role of genomic mutations and the disappearance of immune memory and seroconversion should be investigated.

## Introduction

A number of studies have indicated that the plasma-derived hepatitis B (HB) vaccine is safe, effective, and have documented its use throughout the world since 1980.^1–4^ The HB vaccine was introduced in China since 1984.^5^ Long An County, located within Guangxi Province, has long been regarded as one of the high HB virus (HBV) endemic counties with an HB surface antigen (HBsAg) prevalence of 16.9% in those aged between 1 and 60 years old^4^ and eventually was selected as one of the five study sites for the clinical evaluation of a plasma-derived HB vaccine.^6^ The HB vaccine has been incorporated into the National Immunization Programs in Long An County since 1987 and delivered to newborns; who received three 10-μg doses of plasma-derived HB vaccine at 0, 1, and 6 months of age. A number of studies since then have indicated that the plasma-derived HB vaccine has good efficacy, protection rate, and subsequently decreased the disease burden of HB disease in Long An County.^1,2,7,8^ However, research has found that the antibody to HBsAg (anti-HBs) positivity rate and vaccine effectiveness declining in the cohort and that HBsAg and antibody to HB core antigen (anti-HBc) prevalence increased a year after immunization.^8^ Another study has determined that followed by a gradual decline in concentration of Anti-HBs by year, the Anti-HBs positivity rate in children who developed protective Anti-HBs levels after the primary HB vaccination series dropped from 99% (1 year after vaccination) to 83% (5 years), 71% (7 years), and final to 37% (15–17 years).^9,10^ It is widely known that if the Anti-HBs level falls below 10 mIU/mL, the individual is vulnerable to HBV infection, so a booster dose of HB vaccine is then recommended.^11^ However, other researchers have contended that irrespective of gradual decline and loss of Anti-HBs, adequately performed primary immunization in healthy persons ensures long-term protection against acute and chronic stages of HB.^12^ In fact, T and B lymphocytes, whose responsiveness prevails in the presence of Anti-HBs in serum, are argued to be true markers of immunity. Therefore, research should be confirmed the role of Anti-HBs versus T and B lymphocytes and their roles in producing successful protection and antibody resistance to HB. Despite the current prevailing scientific discussions about the long-term effectiveness of the HB vaccine, it remains unknown whether the HBsAg status of participants with a successful immune response after receiving a full course of primary HB vaccination can change over time, especially 25 years after the primary vaccination has completed. Hence, the aim of this study is to explore HBsAg seroconversion caused by HBV from 2015 to 2019 among those in the long-term immunization cohort in Long An County, Southern China.

## Materials and Methods

Following a serological investigation of the persistence of Anti-HBs to HB vaccine in 2015, we continued a two-phase sampling method in the same immunization cohort of the HB vaccine in July 2019. Blood samples were collected from the participants, and HBsAg, HB e-antigen (HBeAg), antibody to HBeAg (anti-HBe), and Anti-HBc levels were qualitatively tested (comparing positive vs negative results) among the plasma samples; then an HBsAg quantitative detection (measure the numerical level) was carried out for those with HBsAg positivity to record the level of the antigen in the blood, and a consequential HBV-DNA quantitative detection was launched for those with a level of HBsAg higher than 250 IU/mL. We quantitatively tested the level of Anti-HBs in all participants simultaneously. We confirmed the seroconversion of HBsAg if the participants were serum-negative in 2015 but positive in 2019 and classified them as HBsAg seroconversion within the 4-year time period.

### Study setting

Our study was carried out in Long An, a county located in Guangxi, Southern China with 419,000 residents in 2019. Long An implemented a universal immunization program and delivered the HB vaccine to newborns and infants with the support of the World Health Organization (WHO) vaccination campaign beginning in 1987. It is considered as one of the earliest counties to deliver HB vaccine to newborns and infants in a county-territory scale in China.

### The vaccine and immunization schedule

Newborns and infants born from 1987 to 1993 received a full course of primary HB vaccination with each dosage of 10 μg/mL. The immunization followed the 0-, 1-, and 6-month immunization schedules. The vaccine was developed using a sub-viral particle (HBsAg) purified from the inactivated plasma of asymptomatic carriers of HBV (Beijing Biological Products Co., Ltd., Beijing, China).

### Study design and study cohort

The goal of this study was to explore recent HBV seroconversion in a cohort born between 1987 and 1993 who had received a full course of primary HB vaccination. To detect breakthrough infections in the interval from 2015 to 2019, a cross-sectional survey and blood sampling were conducted. First, a face-to-face guided survey including personal demographic characteristics, self-reported behaviors (e.g., tobacco use, alcohol use) and HB among personal contacts were conducted. Blood was then sampled to measure serological biomarkers including HBsAg, Anti-HBs, HBeAg, Anti-HBe, and Anti-HBc levels. We then compared the findings from 2019 with the baseline 2015 results. HBV seroconversion was confirmed if an individual was HBsAg-negative in baseline 2015 but was subsequently positive in2019.

**Figure 1.**
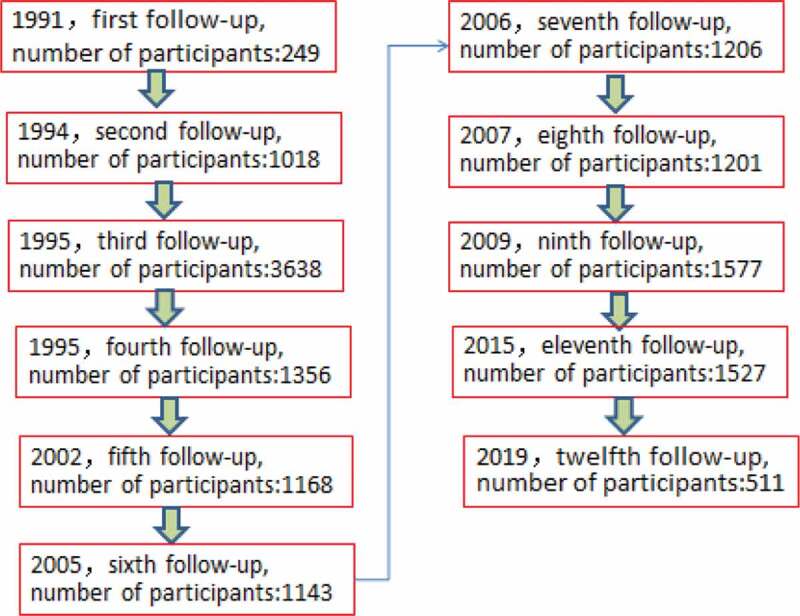
Flow chart for followed-up evolution of the HB vaccine immunization cohort which born in 1987–1993, indicating the times the cohort have been followed up including the year for each follow-up, and the number of participants who completed the follow-up

After the universal immunization program among infants in Long An County, personal demographic and contact information, including name, parents’ name, gender, address, birthday, vaccination date of each dose of HB vaccine was been recorded at completion of the primary vaccination course. Researchers in Guangxi first followed-up the cohort in 1991, A number of individuals were followed-up, with serological biomarkers of HBV measured in a period of time (Figure 1).

### Laboratory assay

Blood samples were tested for five HBV markers in serum – HBsAg, Anti-HBs, HBeAg, Anti-HBe, and Anti-HBc – at Guangxi Ruigu Biological Testing Co. Ltd. Preliminary qualitative testing (positive or negative) of HBsAg, HBeAg, Anti-HBe, and Anti-HBc was performed using enzyme-linked immunosorbent assay (ELISA) (Wantai, Beijing China), and the quantitative testing of HBsAg and Anti-HBs levels were performed using ARCHITECT HBsAg and Anti-HBs Reagent Kits (Abbott Ireland Diagnosis Division, Sligo, Ireland). According to protocols provided by the manufacturer, positive and negative cutoffs were calculated with the positive and negative controls as required by the diagnostic kits. HBsAg >0.05 IU/mL was considered reactive. The minimum and maximum detection limits of Anti-HBs were 2.5 and 1000 mIU/mL, respectively. The detailed experimental method of the survey in 2015 in this cohort was well described in elsewhere.^8^

### Laboratory quality control

Both the qualitative and quantitative tests performed using the reagents with the same batch number. After evaluated the sensitivity and the specificity of five testing reagents in China, we chose the test kits from Wantai Company, Beijing China for the ELISA test, and test kits from Abbott Ireland Diagnosis Division, Sligo, Ireland for the quantitative test. First, strict testing procedures were established, and an experimental platform was set up which subsequently become a routine test platform in the company. Second, the relevant equipment has been adjusted before the qualitative and quantitative tests performed. Third, the standard testing curve was established using HBsAg produces with different standard levers of concentration. Fourth, set up positive and negative control, according to the test protocol, two positive and three negative controls were set on each enzyme plate, which allowed the technicians easily to identify the enzyme plate with abnormal reaction. Technicians will repeat the test once the cutoff values of the samples were within three standard-deviations.

### Statistical analysis

Data were analyzed using R software (version 3.6.1, R Foundation for statistical computing, Vienna, Austria) and SPSS (version 23.0, IBM, Armonk, New York, U.S.A). Comparison of those HBsAg positive by gender (male and female) was analyzed by Chi-square test Chi-square statistics, significance level was set at 0.05, and all hypothesis tests were two-sided.

### Ethics

This study was reviewed and approved by the Institutional Review Board (IRB) of the Guangxi Zhuang Autonomous Region Center for Disease Control and Prevention. Written informed consent was obtained from each participant for personal information and blood sample collection immediately before the study.

## Results

### Social demography of participants and baseline Hep B antibody

A total of 1,527 participants born between 1987 and 1993 received a full course of primary HB vaccination and were initially recruited into the study in 2015, and 511 (33.5%) participants were enrolled in the 2019 round of data collection. Data were linked between a total of 143 participants in both 2015 and 2019. Of the 511 participants, 395 (77.3%) were male and 116 (22.7%) were female, with a male-to-female ratio of 3.40:1; the mean age of the participants was 29.49 ± 1.85 (x ± σ) years (range: 25–33 years). Those who reported their occupation as being farmers and those of the Zhuang ethnicity were the majority (Table 1).

**Table 1.** Serological marks of HBV among different social demography in Southern China (n = 511)

	Anti-HBs						
Items	≥10mIU/ml n (%)	<10mIU/ml n (%)	HBsAg Positive n (%)	HBeAg Positive n (%)	Anti-HBe Positive n (%)	Anti-HBc Positive n (%)	n (%)
Total Participants	192 (37.6)	319(62.4)	27(5.3)	8(1.6)	40(7.8)	53(10.4)	511(100.0)
Age							
26 Y	13(42.0)	18(58.1)	1(3.2)	0(0.0)	0(0.0)	2(6. 5)	31(6.1)
27 Y	19(33.9)	37(66.1)	2(3.7)	2(3.6)	2(3.6)	3(5.4)	56(11.0)
28 Y	21(31.3)	46(68.7)	3(100.0)	2(3.0)	2(3.0)	4(6.0)	67(13.1)
29 Y	38(40.0)	57(60.0)	5(5.3)	1(1.1)	9(9.5)	10(10.5)	95(18.6)
30 Y	34(35.1)	63(65.0)	5(5.2)	1(1.0)	7(7.2)	8(8.3)	97(19.0)
31 Y	30(40.0)	45(60.0)	4(5.3)	1(1.3)	10(13.3)	8(10. 7)	75(14.7)
32 Y	29(39.2)	45(60.8)	2(2.7)	0(0.0)	7(9.5)	7(9.5)	74(14.5)
33 Y	8(50.0)	8(50.0)	1(6.3)	1(6.3)	3(18.8)	3(18.8)	16(3.1)
Gender							
Male	152(38.5)	243(61.5)	21(5.3)	5(1.3)	33(8.4)	42(10.6)	395(77.3)
Female	40(34.5)	76(65.5)	6(5.2)	3(2.6)	7(6.0)	11(9.5)	116(22.7)
Education							
Junior high school	122(35.9)	218(64.1)	21(6.2)	6(1.8)	34(10.1)	43(12.8)	340(66.5)
Illiterate	0(0.0)	2(100.0)	0(0.0)	0(0.0)	0(0.0)	0(0.0)	2(0.4)
Elementary school	31(77.5)	9(22.5)	1(2.5)	0(0.0)	1(2.5)	1(2.5)	40(7.8)
High school	48(49.08)	50(51.0)	4(4.1)	2(2.0)	2(2.0)	5(5.1)	98(19.1)
College university and above	13(41.9)	18(58.1)	1(3.2)	0(0.0)	3(9.7)	4(12.9)	31(6.1)
Occupation							
Farmer	105(35.4)	192(64.6)	13(4.4)	4(1.4)	21(7.1)	22(7.4)	297(58.1)
Worker	30(50.0)	30(50.0)	3(5.0)	1(1.7)	4(6.7)	6(10.0)	60(11.7)
Government employee	2(50.0)	2(50.0)	0(0.0)	0(0.0)	0(0.0)	0(0.0)	4(0.8)
Medic	1(33.3)	2(66.7)	0(0.0)	0(0.0)	1(33.3)	0(0.0)	3(0.6)
Public service	7(46.7)	8(53.3)	0(0.0)	0(0.0)	0(0.0)	1(6.7)	15(2.9)
Farmer worker	10(40.0)	15(60.0)	2(8.0)	2(8.0)	6(24.0)	7(28.0)	25(4.9)
Others	37(34.6)	70(65.4)	9(8.4)	1(0.9)	8(7.5)	12(11.2)	107(20.9)

Of 143 participants who involved in both 2015 and 2019, 33.6% (48/143) individuals were with protective Anti-HBs level (≥10 mIU/mL) in 2015. Among those with protective Anti-HBs level, the Anti-HBs mean geometric titer (GMT) was 11.03 mIU/mL (95% confidence interval [CI]: 8.35–28.61) (Table 2).

**Table 2.** Statuses of Anti-HBs, Anti-HBc, HBeAg and Anti-HBe in both 2015 and 2019 for participants in Long An, Southern China (n = 143)

Serological marks	Serological results in 2015		Serological results in 2019	
	n	%	n	%
Anti-HBs				
Positive	48	33.6	47	32.8
Negative	95	66.4	96	67.2
Anti-HBc				
Positive	33	23.1	11	7.7
Negative	110	76.9	132	92.3
HBeAg				
Positive	4	2.8	2	1.4
Negative	139	97.2	141	98.6
Anti-HBe				
Positive	6	4.2	9	6.3
Negative	137	95.8	134	93.7

### Persistence of Anti-HBs and prevalence of HBeAg, Anti-HBe, and Anti-HBc

No HB booster vaccine was given 26–33 years after the primary HB vaccine of three doses in infancy (as recorded on the immunization card); the positive seroprotection rate was 37.6% among the 511 participants. A total of 3.3% of participants were found to have a Anti-HBs concentration >1000 mIU/mL. The Anti-HBs GMT of participants was 11.1 mIU/mL (95% confidence interval [CI]: 8.51–30.72).

**Figure 2.**
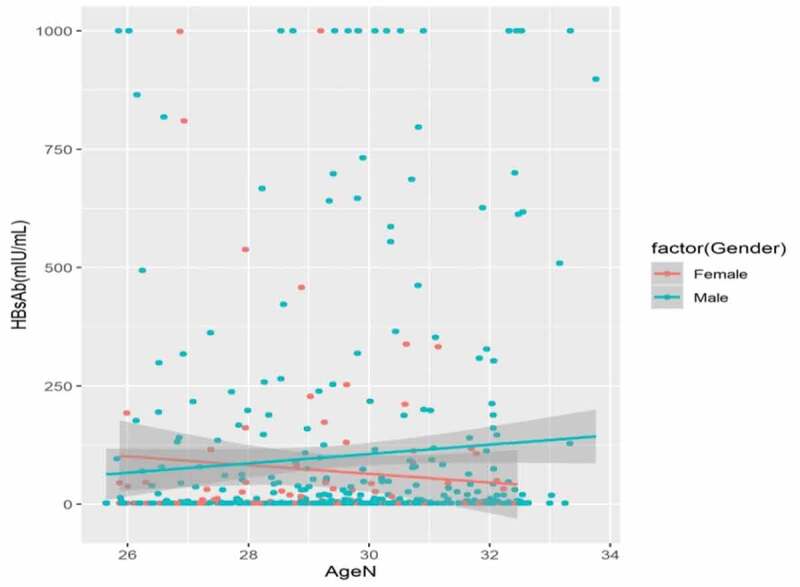
Dot-plot of Anti-HBs concentration (mIU/ml) with 95% CI among male (green dots) (n = 395) and female (red dots) (n = 116) born in 1987–1993 year, Long An County, Southern China (n = 511)

Of the 511 participants, the overall prevalence of HBeAg, Anti-HBe, and Anti-HBc was 1.6% (8/511), 7.8% (40/511), and 10.4% (53/511), respectively (Table 1 and Figure 2).

### The prevalence of HBsAg and seroconversion

Of the 511 participants, 27 (5.3%, 27/511) were HBsAg positive; of those with HBsAg positivity, 6 participants were female (5.2%, 6/116), and 21 were male (5.3%, 21/395); the HBsAg positivity rate was no significant difference between males and females (*P* = .4093). Among the 1527 participants involved in study in 2015, HBsAg prevalence was 3.5% (53/1527). Of the 143 participants involved in both studies in 2015 and 2019, 5 (3.5%, 5/143) seroconversions of HBsAg were observed between 2015 and 2019. Among those with protective Anti-HBs level, 2 (4.2%, 2/48) participants were HBsAg seroconverted; of those without protective Anti-HBs level, 3.2% (3/95) were with HBsAg seroconversion. Participants C and D exhibited a successful immune response with Anti-HBs concentration of 112.04 mIU/ml and 81.38 mIU/ml in 2015, respectively, while participants A, B, and E did not show a successful immune response in 2015 (Table 3).

**Table 3.** Individuals of HBsAg seroconversion in 2015–2019 of the same cohort in Long An, Southern China (n = 5)

	Serological results in 2015				Serological results in 2019		
Individuals	Qualitative result of HBsAg	Qualitative result of Anti-HBs	Concentration of Anti-HBs (mIU/ml)	Qualitative result of anti-HBc	Qualitative result of HBsAg	Concentration of HBsAg (IU/ml)	Concentration of HBV-DNA (IU/ml)
Participant A	Negative	Negative	0.0	Positive	Positive	1.8	---
Participant B	Negative	Negative	0.4	Negative	Positive	1.3	---
Participant C	Negative	Positive	112.0	Negative	Positive	> 250.0	364000000
Participant D	Negative	Positive	81.4	Negative	Positive	> 250.0	2510
Participant E	Negative	Negative	0.0	Negative	Positive	2.2	---

## Discussion

This two-phase sampling study found that of 143 participants enrolled in studies in 2015 and 2019. Across this time, HBsAg seroconversion occurred among five (3.5%) participants. This is consistent with other research as several studies have shown that the rate of breakthrough infection to be low among vaccine recipients, and that they have mainly manifested as Anti-HBc-positive (1.0–13.8%), transient HBsAg-positive (0.7–5.4%), or HBV-DNA-positive (0.2–0.9%).^11–13^ However, the HBsAg seroconversion in this study can be considered higher than expected as the duration between data collection was a 4-year period, the high prevalence of HBV in Long An, the disappearance of immune memory of T lymphocytes, HBV genomic mutations, or the gradual decline and loss of Anti-HBs may play a role in such a higher seroconversions. Additionally, HBsAg seroconversion was observed among individuals who had a protective Anti-HBs concentration level 4 years earlier, which may present a new understanding of the possibility of HBsAg seroconversion 26–33 years after vaccination. Multiple checking methods implemented in this study have guaranteed a reliable result. First, participants received an ID check before their bloods were sampled; only the participants whose ID information matches the information in the cohort registration tables were allowed to access the investigation and the blood sampling. Second, standard methods of laboratory quality control including strict testing procedures, equipment adjusted, establishment of standard testing curve, and building of positive and negative control to ensure the correct samples, and the detection. However, future research is needed as the mechanisms underlying these seroconversions are currently unknown.

With the introduction of universal HB immunization, the prevalence of HBsAg in China adjusted for people aged 1–59 years declined from 9.8% in 1992 to 7.2% in 2006.^14^ The weighted HBsAg prevalence among people aged 1–29 years declined from 10.1% in 1992 to 5.5% in 2006 and to 2.6% in 2014, which changed China from a classification as a highly endemic country to one that has an intermediate endemic rate.^15^ However, a number of studies have shown that the prevalence of HBsAg in Guangxi (the province in which this research was conducted) continues to be higher, and has not seen the same improvements that have been seen across the rest of China. In 2019, the weighted HBsAg prevalence among people aged 1–59 years was 7.2% in Guangxi, China,^16^ which was similar to that in 2006 in China, while the prevalence was 9.3% among people aged 15–59 years old.^16^ The study conducted among participants born in 1987–1993 in 2015 in Long An County indicated that the HBsAg positive rate was 3.5%;^8^ meanwhile, another study performed among people born from 1987 to 1989 in Long An in 2016 indicated that the HBsAg prevalence was 4.9%.^17^ Both the HBsAg positive rates were lower than the rate (5.3%) reported in the present study. The increasing trend of the HBsAg positive rates in this cohort over time indicated that the breakthrough infections do occur, and it should not become an ignored phenomenon to suggest that a booster dose of HV vaccine is needed after 25 years of receiving primary 3-dose vaccination series.

Researchers believe that adequately performed primary immunization in healthy persons ensures long-term protection against acute and chronic HB because anamnestic response to a booster injection of HB vaccine has been confirmed by a number of studies, which means vaccine protection through immune memory of cellular response following HB vaccination persists longer than humoral response.^18–20^ These studies demonstrated high rates of anamnestic response to booster doses, and even undetectable Anti-HBs levels can provide strong immune reactions with rapidly increasing geometric mean concentration (GMC). However, results from another investigation showed that the anamnestic response rates after receiving a booster dose of vaccine were 85.3% (64/75, year 10) vs. 73.6% (39/53, year 15).^21^ This evidence indicated that the immune memory of T lymphocytes would decrease and eventually wear off over time; thus, HBsAg seroconversion might occur among individuals who experience decline of immune memory.

HBsAg mutants have an association with major clinical and biological impacts such as failure to detect HBsAg, development of hepatocellular carcinoma (HCC), and immune escape. The structure of HBsAg is well described elsewhere,^22^ with the dominant epitopes of HBsAg that reside in the “a” determinant of the major hydrophilic region. The nucleotide mutation on both loops of the “a” determinant might play an important role in the lack of protection of antibodies produced by the vaccination; thus, the occurrence of HBV infection in individuals with a previous successful immune response can be observed. Researchers in Italy reported that children with a strong immune response to the HB vaccine have been infected by HBsAg-positive mothers or with other HBsAg-positive household close contacts.^23,24^ A more detailed analysis identified an association of vaccine escape with a point mutation from glycine to arginine at position 145 (G145R).^25^ This G145R mutation has confirmed the most frequently detected vaccine-escape mutant.^23,24,26-28^

## Limitations

This study has some limitations. First, the number of participants involved in both phases of sampling was small, which may bias the representative percentage of HBsAg seroconversion in the 26–33-year vaccination cohort. Furthermore, the work of molecular detection and sequencing of the determinant of HBsAg for the five HBsAg-positive participants to identify potential HBsAg mutants hasn’t done yet, and the study is also limited to a descriptive analysis of a population-based cross-sectional study and it is not possible to derive correlations between mutations and the breakthrough infection. The molecular detection and sequencing of the determinant of HBsAg for the five HBsAg-positive participants are an important future direction of this study.

## Conclusions

Despite these limitations, this study provides important indications for clinical practice future research. For clinical practice, recommendations promoted worldwide suggest more than 20-year lasting protection against HB following primary immunization. This study found that 3.5% of participants in this cohort seroconverted 26–33 years after the primary vaccination, and confirmed the individuals whose HBsAg statuses have been converted. The finding is unique because less study has reported the seroconversion rate of HBsAg within a specific time period, and less study has successfully pointed to the individuals whose HBsAg status have been converted. Additional analysis is required for the risk factors related to the seroconversion, such as information of HBsAg status in the close contacts and genomic mutation at a special position.

This study detected serological HBsAg seroconversion among a cohort of participants than had protective Anti-HBs levels 4 years earlier in a high HBV epidemic region. The role of genomic mutation or the disappearance of immune memory of T lymphocytes in HBsAg seroconversion should be considered by future research. Additional analysis is required to understand the risk factors related to the seroconversion, such as information of HBsAg status in the close contacts, genomic mutation at special position, and others. HBsAg seroconversion should be taken into account by vaccine manufacturers and health policy makers to ensure consistent progress toward achieving the target of eliminating hepatitis B as a worldwide problem by 2030.
